# Evaluation of maternal performance about food security in dietary diversity for children aged 12-24 months and its relationship with anthropometric measurements

**DOI:** 10.1186/s12887-023-04070-6

**Published:** 2023-05-23

**Authors:** Sedigheh Yeganeh, Niloofar Motamed, Saeid Najafpour Boushehri, Razieh Bagherzadeh, Maryam Ravanipour

**Affiliations:** 1 School of Nursing, Gerash University of Medical Sciences, Gerash, Iran; 2 Department of Nursing, School of Nursing and Midwifery, Bushehr University of Medical Sciences, Bushehr, Iran; 3 Department of Community Medicine, School of Medicine, Bushehr University of Medical Sciences, Bushehr, Iran; 4 Department of Nutrition, School of Health and Nutrition, Bushehr University of Medical Sciences, Bushehr, Iran; 5 Department of Midwifery, School of Nursing and Midwifery, Bushehr university of Medical Sciences, Bushehr, Iran; 6 Department of Nursing, School of Nursing and Midwifery, The Persian Gulf Tropical Medicine Research Center, The Persian Gulf Biomedical Sciences Research Institute, Bushehr university of Medical Sciences, Bushehr, Iran

**Keywords:** Anthropometric measurements, Complementary Feeding, Food Security, Maternal Performance

## Abstract

**Background:**

Despite growing awareness of the problem of food security, some areas of Iran continue to experience food insecurity. The aim of the present study was to evaluate maternal performance about food security in dietary diversity for children aged between 12-24 months and its relationship with anthropometric measurements in Bushehr.

**Methods:**

A cross-sectional study was carried out using 400 mothers of children aged from 12 to 24 months in Bushehr selected via quota sampling. Data were collected using a reliable localized version of a 32-item food frequency questionnaire, consisting of six subscales, with a Cronbach's α: 0.81. The anthropometric measurements of height and weight were also calculated. Data analysis was performed using median, Mean±SD and multinomial logistic regression test, and odds ratio in SPSS, version 18.

**Results:**

According to standard servings, only 24% of the mothers fed their infants cereals, whereas 54.8%, 36.3%, 39.8%, and 20.3% of the mothers used meat, fruits, vegetables, and dairy products, respectively. The strongest associations were between attendance at educational classes and vegetable consumption (OR=2.09, CI=1.03-4.21), age at the onset of complementary feeding and the consumption of meat (OR=1.30, CI=1.02-1.66) and fruits (OR=1.44, CI=1.03-2.03), and the mothers’ level of education and use of dairy products (OR=0.29, CI=0.09-0.90). No evidence of a significant association was found between consumption of any food groups and the anthropometric measurements.

**Conclusions:**

Mothers in Bushehr performed poorly in providing their infants with the required nutrition with regard to dietary diversity and amount of food. However, their performance can be improved by enhancing their basic nutrition knowledge, holding practical classes on food preparation for them, and focusing on mothers with infants in high-risk groups, e.g. infants suffering from excessive weight gain, obesity, and malnutrition.

## Background

Subsequent to the Universal Declaration of Human Rights, improving the nutritional status, especially of mothers and children, has become a major concern in all countries [1]. Food security is defined as a condition in which all people have physical, social, and economic access to sufficient amounts of nutritious food on a regular basis [2]. In recent years, there has been an increase in awareness about food security in Asia [3]. However, for various reasons, Iran is still dealing with food insecurity: food insecurity is widespread throughout this country according to studies [4, 5].

According to reports on food security, Bushehr is among the cities suffering from serious food insecurity [6, 7]. Previous research has reported a high level of food insecurity among mothers and children in Bushehr, and infants are at the highest risk [7]. Food insecurity among infants is associated with physical, developmental, and behavioral problems. Particularly during the first two years of life, it can increase the risk of diseases that may lead to mental and behavioral dysfunction in adulthood [8–10]. Hence, complementary feeding must supplement breastfeeding and provide infants with the required level of nutrition [11]. According to the latest statistics, malnutrition accounts for at least 35% of child mortality under the age of 5 years, 6% of which could be prevented with proper complementary feeding [12]. Mothers, as the key providers of household food security, play an important role in the healthcare and nutrition of infants. Their performance in the area of nutrition is directly associated with food security for their infants, particularly during the complementary feeding period [13, 14].This period is from 6 months to 2 years of age, during which time infants’ access to food and the manner of being fed depends on the quality of their mothers’ feeding practices and behaviors [15] .

There are various methods for assessing food security, among which determination of food frequency and measurement of anthropometric indexes are the most common. Food frequency is extensively used for the long-term evaluation of food intake and to predict the health status of people [16] . It is influenced by various factors, such as culture, belief, social status, geographical location, regional dietary pattern, and the availability of food resources [17].

Food insecurity is associated with availability of food, cultural characteristics, social issues, and place of residence. These parameters play an important role in food selection, preparation, and storage. Additionally, any type of intervention requires basic knowledge about maternal performance and the nutrition of children. However, there are few studies on mothers’ performance and food frequency in developing countries. Despite the gravity of food insecurity in Bushehr, no studies have been conducted to address this issue. Consequently, the present study evaluated maternal performance about food security in dietary diversity for children between 12-24 months and its relationship with anthropometric measurements.

## Methods

The present study is a part of a larger descriptive (quantitative) and analytical work of research with a cross-sectional design conducted in 2016 in Bushehr, Iran. Bushehr is the capital of Bushehr province in southern Iran. This region has a warm and humid climate for most of the year.

The sample size was calculated in accordance with the results of similar studies on food awareness, performance [18] and food security in Iran [19] and using the *n*=Z^2^_1-α/2_ P(1-P)/d^2^ formula. A total of 400 individuals were selected from all the ten comprehensive health service centers in Bushehr using the quota sampling method. The inclusion criteria were: being a mother with an infant aged 12-24 months, absence of psychological disorders and chronic musculoskeletal diseases according to a self-report statement and medical files, having an infant with a birth weight of between 2.5-4 kg, having initiated complementary feeding after 6 months of age, absence of diagnosed chronic diseases in neonates affecting their appetite and feeding, and absence of acute gastrointestinal diseases in the months prior to the start of the study. The data collection tool was a researcher-made questionnaire. The questionnaire was completed by the mothers after they were informed of how it worked. The illiterate participants had their questionnaires filled in by the researcher, based on their responses to the items, following a clear explanation of each item.

### Data collection

Data were collected using a specific demographics survey and a localized food frequency questionnaire. The researchers also determined the infants’ anthropometric measurements. A specific 23-item demographics survey was developed using the findings of a previous study and the required demographic variables of the present research. Demographic characteristics of the mothers included age, number of children, and the kind of childcare they used (center-based or home-based daycare). Additionally, the infant’s characteristics, including birth weight, age, birth order, and age at first complementary feeding, were recorded. The measured anthropometric indexes, in accordance with those introduced by the WHO and Iranian Ministry of Health [20, 21], were: weight-for-age (WFA), height-for-age (HFA), and weight-for-height (WFH). The weight-for-age index (WFA) was divided into three groups: underweight (− 3 ≤ z-score < − 2), normal (− 2 ≤ z-score ≤ + 1), and overweight (above + 1). The height-for-age (HFA) index was divided into four groups: very short height (less than − 3 z-score), short height (− 3 ≤ z-score < − 2), normal (− 2 ≤ z-score ≤ + 3), and tall (higher than + 3). The weight-for-height index (WFH) was divided into 5 groups: thin (less than − 2), normal (− 2 ≤ z-score ≤ + 1), risk of overweight (+ 1 < z-score ≤ + 2), overweight (+ 2 < z-score ≤ + 3), and obese (> + 3).

The design and assessment of the psychometric properties of the localized food frequency questionnaire (lFFQ) for food security measurement were based on Waltz method [22]. To design this questionnaire, the researchers reviewed different types of questionnaires, among which the Harvard FFQ, which reports on the consumed food over the past month, was selected [23]. The main reason for selecting this questionnaire was that children regularly follow behavioral patterns in complementary feeding and may not consume a particular food group daily [24, 25]. Moreover, the questionnaire provides useful information on food problems through periodic evaluations [26]. The items of the questionnaire were localized to incorporate the usual diet of families in Bushehr, particularly during the complementary feeding period. Accordingly, six food groups were defined, namely grains and cereals (7 items), meat and meat products (6 items), fruits and fruit juices (7 items), vegetables (3 items), dairy products (8 items), and nuts (1 item). These groups were listed in a tabular format with specified servings for infants aged 1-2 years (e.g., 45 gm. of fish equals three tablespoons) and scored based on 8 scales according to the mothers’ use of each group to feed their infants in the past month (2-3 times a day: 90 points, once a day: 30 points, 3-4 times a week: 12 points, once a week: 4 points, 2-3 times a month: 3 points, once a month: 1 point, rarely: 0 points, and never: 0 points). The participants were requested to mark the column related to the nutritional intake of their infants. For instance, the mothers were asked to mark a scale based on how often their infants consumed ½ cup rice. The daily servings of food were calculated by dividing the total score by 30 days. The adequacy of dietary diversity was measured according to the minimum and maximum food servings: 4-6 portions of cereals, 1-2 portions of fruits, vegetables, and meat (each), and 2-3 portions of dairy products.

The face validity of the questionnaire was assessed with 10 mothers of infants aged 1-2 years with different levels of education. The face validity coefficient was >1.5 for all the items in the questionnaire. The content validity was evaluated by 12 experts on pediatric nutrition (nutritionists, community medicine specialists, nurses, and public health education specialists). The content validity ratio for each item was >0.81. The reliability of the questionnaire was examined with 30 eligible mothers and the Cronbach’s alpha was found to equal 0.81. Hence the validity and reliability of the questionnaire were confirmed. The LFFQ was scored in the following manner: daily consumption of cereals per serving (unit) as suggested by the questionnaire fell into three categories: less than 4 units=insufficient, 4-6 units=enough, and more than 6 units=too much; likewise, daily consumption of meat, fruit, and vegetables fell into three categories: less than 1 unit=insufficient, 1-3 units=enough, and more than 3 units=too much; daily consumption of dairy products was also divided into three categories: less than 2 units=insufficient, 2-3 units=enough, and more than 3 units=too much. Being placed in any category except “enough” indicated the maternal’s unsatisfactory performance (“too much” or “insufficient”). This questionnaire did not have a total score and the mothers’ performance for each food group was assessed separately.

### Data analysis

The data were analyzed using SPSS (version 18.0). logistic regression analysis were used to determine the relationship between the anthropometric measurements (WFH, WFA, HFA) and the maternal performance with regard to food security as determined by the lFFQ questionnaire. The multinomial logistic regression was used to establish the association between the food groups and the participants’ demographic characteristics. The odds ratio was calculated based on a 95% confidence interval. The descriptive data (demographic characteristics and anthropometric measurements) were presented as median and Mean±SD. P<0.05 was considered as statistically significant.

### Ethical considerations

The study was approved by the ethics committee of Bushehr University of Medical Sciences (Bushehr, Iran), as also described in our previous studies [21, 27]. The participants of the present study were informed about the goals of the research, the methodology, and confidentiality of their information. Written informed consent was obtained from all the participants.

## Results

The demographic characteristics of the participants showed that 81% of the mothers were housewives and the rest of them worked outside the home. The mean age of the participants was 29.53±4.92 years (range 17-45 years). Most of the participants had a high school diploma (41%) or a university degree (43.3%). At the time of the research, 54.5% of the participants lived in a home they owned, 37.8% lived in rented accommodations, and 7.5% lived with a relative. The mean birth order of the infants was 1.72±0.75 (range 1-6), and 53.8% of them were girls. The mean age of the infants was 16.44±3.96 months and complementary feeding started at the mean age of 6.25±0.88 months. The average number of children in the families was 1.77±0.78 (range 1-6). A detailed description of the demographic characteristics has been presented in the researchers’ previous study [27].

**Fig. 1:**
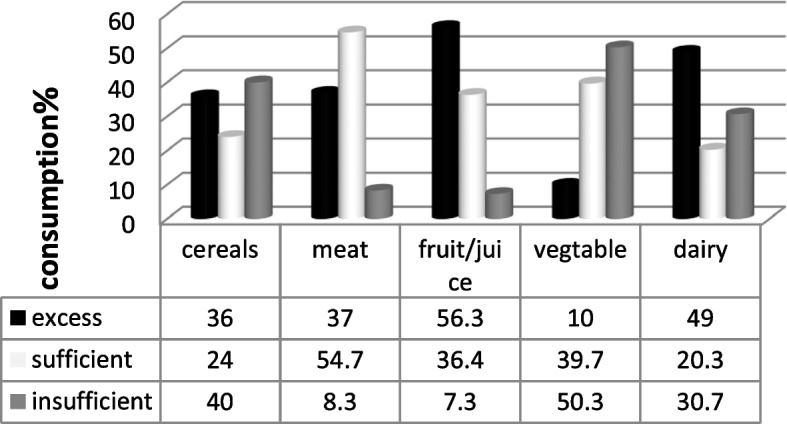
An overview of maternal daily performance of complementary feeding with respect to food groups. Cereals: 4-6 servings; Vegetables, fruits, and meat: 1-2 servings; Dairy products: 2-3 servings

In terms of maternal performance in children between 12-24 months during the complementary feeding period, 40% of the infants consumed grains and cereals (rice, wheat, barley, bread, spaghetti, biscuits, cakes, and potatoes), less than the standard daily servings. Meat and meat products (red meat, chicken, eggs, fish, and shrimps) were adequately consumed by 54.7% of the infants. The consumption of fruits and fruit juices (citrus fruits, apples, peaches, bananas, cherries, mangos, pineapple, dates, and date products) in 56.3% of the infants was above the standard daily servings. Furthermore, 50.3% of the infants consumed vegetables (raw or steamed) less than the recommended servings. Dairy products (milk, dried milk, yogurt, cheese, ice cream) were consumed above the standard amounts by 49% of the infants (Fig. 1). Fruits, vegetables, and dairy products were not consumed by 0.5%, 4%, and 1.3% of the infants, respectively. In addition, 25.9% of the infants were only fed formula milk, 12.3% were fed both formula milk and breast milk, while 11.5% consumed neither.

With regard to anthropometric measurements, the results showed that 71.5% of the children had normal weight, 26% were overweight to obese, and only 2.5% had weight loss. The mean weight of the children was 10.47 ± 1.66 kg (range 7.50-18.91). The mean of HFA was 80.16±4.82 cm and normal and short heights were registered in 93.5% and 4.6% of the infants respectively. As for weight-for-height index, 72.75% had a weight for normal height and the remaining 27.25% were at risk of becoming overweight to obese. Based on logistic regression analysis, there was no evidence of a significant association between the amount of dietary diversity (in all food groups) and the anthropometric measurements (Table 1).

**Table 1:** The results of logistic regression analysis of the association between dietary diversity and anthropometric measurements in infants aged 12-24 months (*n*=400)

Variables		HFA (normal-tall)^a^			WFA (underweight-normal) ^b^			WFH (wasted-normal-risk of overweight)^c^		
		OR	CI	P	OR	CI	P	OR	CI	P
Cereals	Insufficient	1.20	0.29-4.94	0.79	0.86	0.48-1.54	0.63	1.12	0.43-2.92	0.81
	Sufficient	1			1			1		
	Excessive	2.06	0.54-7.83	0.28	1.00	0.55-1.78	1.00	0.78	0.26-2.13	0.58
Meat	Insufficient	2.33	0.59-9.10	0.22	0.93	0.39-2.18	0.87	1.75	0.54-5.59	0.34
	Sufficient	1			1			1		
	Excessive	0.98	0.34-2.83	0.97	1.07	0.67-1.73	0.75	0.72	0.30-1.74	0.47
Fruits	Insufficient	1.46	0.28-7.41	0.64	0.86	0.34-2.18	0.75	1.40	0.36-5.38	0.62
	Sufficient	1			1			1		
	Excessive	0.81	0.29-2.24	0.69	0.93	0.58-1.50	0.79	0.80	0.35-1.82	0.60
Vegetables	Insufficient	1.96	0.67-5.67	0.21	0.88	0.55-1.42	0.62	0.91	0.41-2.04	0.83
	Sufficient	1			1			1		
	Excessive	0.79	0.9-6.95	0.83	0.75	0.33-1.72	0.50	0.64	0.13-3.00	0.57
Milk	Insufficient	2.38	0.48-11.77	0.28	0.85	0.46-1.58	0.62	0.75	0.24-2.33	0.62
	Sufficient	1			1			1		
	Excessive	1.90	0.40-8.99	1.90	0.66	0.37-1.18	0.17	1.03	0.38-2.77	0.94

Significance P≤0.05

*OR* Odds ratio, *CI* Confidence Interval (95%)

^a^ ≥ −2 Z-score

^b^ ≤ + 1 Z-score

^c^ < + 2 Z-score

The association between the food groups and demographic characteristics was assessed using the multinomial logistic regression analysis. For comparison purposes, the standard serving in each group was regarded as the baseline. The association between the consumption of cereals and demographic characteristics was not statistically significant. Attendance at educational classes was associated with vegetable consumption (OR=2.09, CI=1.03-4.21), age at the onset of complementary feeding and the excessive consumption of meat (OR=1.30, CI=1.02-1.66) and fruits (OR=1.44, CI=1.03-2.03), and the mothers’ level of education and use of dairy products (OR=0.29, CI=0.09-0.90). However, no evidence of a significant association was found between consumption of any food groups and the anthropocentric measurements (Table 1).

## Discussion

The present study was conducted to evaluate maternal performance about in dietary diversity for children aged 12-24 months and its relationship with anthropometric measurements. The results indicated that more than 50% of the mothers in Bushehr performed unsatisfactorily in providing their infants with acceptable dietary diversity from all food groups. The only exception was meat, which was used by slightly more than 50% of the mothers. It can be assumed that the main issue was the mothers’ lack of practical knowledge about food for infants, despite having a good academic education and general knowledge of complementary feeding [27]. This indicated that, although Iran has made major improvements in educating mothers about complementary feeding (education provided by comprehensive health centers), the feeding behaviors of the mothers is still unsatisfactory, particularly beyond the infants’ first year of age. May be, the complementary feeding education given in the health centers mainly focused on the onset of complementary feeding and infants’ readiness, and less attention may be given to their needs beyond the first year of age. It seems nutrition education is not given at Iranian schools, nor does Iran have special centers that provide mothers with practical training on nutrition. In a study by Farivar et al. [28], the mean level of knowledge among the people in Bushehr about the role of different food groups was 50%. Shuo Wang (2021) reported that infants aged 12-23 months were not fed according to recommended nutrition standards [29]. In line with the results of the present study, Beshadu Bedada Feyisa et al. reported that vegetable intake was sufficient only among 37.8% of infants aged 6-23 months in Southern Ethiopia [30]. Despite the importance of vegetable consumption in a child’s health and in prevention of chronic diseases [31], the intake of vegetables by children in Bushehr was only 1-3 times weekly. Such deficiency could be due to the short shelf life of vegetables, the misconception that children cannot digest vegetables, or the fear of parasitic contamination by raw vegetables. In a systematic review, Hendrie et al. (2016) proposed measures to increase vegetable consumption among children. They recommended enhancing the availability of vegetables in the market, encouraging vegetable consumption by families, and providing the necessary education and social support [32].

One of the highly consumed food groups in complementary feeding is cereals, but the performance of the mothers in Bushehr in feeding cereals to their infants was unsatisfactory. Some of the cereals commonly consumed by children in Bushehr were found to be rice and wheat bread. Their intake of other cereals, such as barley and ready-to-eat cereals (e.g., corn flakes), was significantly lower. This could be due to lack of access to these foods, poor knowledge about their benefits (as a suitable breakfast), or high costs. Michelle Klerks (2019) considered it necessary to introduce cereals into complementary feeding [33].

Dairy products, as well as breast milk, form another important food group during the complementary feeding period and play an important role in the growth of children. Moreover, in Islam, breastfeeding is strongly stressed by the Islamic culture, beliefs, and practices [34, 35]. Among the Iranians, the traditional belief is to breastfeed infants for two full years with a 2-month difference between male and female infants. Some mothers tend to breastfeed their infants beyond the first year and consider breast milk as the main food source for their infants. In the absence of solid food and irregularity in feeding times, formula milk is used. Iranian health centers recommend the consumption of food commonly consumed by adults in Iran as suitable for infants older than 1 year [36].

In the present study, the researchers did not find any significant associations between the anthropometric measurements and the consumption of food groups by infants (i.e., the maternal feeding practices). In contrast, in their study, Thaweekul et al. (2021) reported a negative association between the infant and child feeding index and nutritional status [37]. However, in line with the findings of the present study, a study of 1,816 children under five years old by Joe et al. (2019) reported a very insignificant association between anthropometric measurements and nutritional failure among the children [38]. These discrepancies could be explained by the fact that the present study focused on the main food groups for the evaluation of maternal performance, while other foods (e.g., fat, snacks, and fast food) might also have been consumed by the infants. Furthermore, anthropometric measurements are affected not only by nutrition, but also by such parameters as genetics, environment, climate, and physical activity. The lack of an estimation of the calorie intake and level of physical activity by the infants could also have contributed to the difference between the findings.

The results of the study showed that the risk of excessive weight gain and obesity among the infants aged 12-24 months was 26% (i.e., one in four of the population). Based on previous studies, excessive weight gain and obesity can continue into adolescence, and obese adolescents are likely to become obese adults [39]. The stigma of obesity is one of the problems in long-term childhood obesity and can cause psychosocial damage in the future of obese infants [40]. Jones et al. (2017) reported that the level of obesity in European children aged >5 years was 28.6% [41]. The guiding principles for feeding infants, in addition to measures for prevention of undergrowth and emaciation, are associated with excess weight, obesity, and imbalanced nutrition. Appropriate dietary habits and education of parents are the key factors in ensuring a healthy food regimen during both childhood and adulthood [42]. Considering the high overweight/obesity rate (26%) in Bushehr, implementation of such intervention programs, particularly for the mothers of infants aged 12-24 months, is essential.

## Conclusion

To the best of our knowledge, this is the first study that evaluated maternal performance in food security in Bushehr using a localized questionnaire, which allowed for cultural sensitivities. Mothers in Bushehr performed poorly in providing their infants with the required nutrition according to dietary diversity and amount of food. It is strongly recommended that the mothers of infants in Bushehr should be educated in complementary feeding to achieve the required level of food security for infants and children. This education could be in the form of an educational booklet, taking the local cuisine into account, specifying the necessary quantity of the servings of each food type, how to estimate food portions, and how to properly prepare and store food. To determine the cause of the prevalent weight gain among infants in Bushehr, further studies are required on how parents obtain their nutritional information and there is need for an accurate measurement of food consumption, both in the form of micro- and macro-nutrients, as well as whole foods.

### Limitations

The main limitation of the present study was the mothers’ lack of understanding of some items in the questionnaire, despite extensive explanations provided by the researchers. They had inadequate knowledge about the quantity of the servings of each food type, and at times could not remember the food groups. This may have adversely affected the accuracy and transferability of the results. In addition, by excluding other foods (e.g., fat, snacks, and fast food), the researchers could not evaluate their association with anthropometric measurements.

## Data Availability

The datasets used and/or analyzed during the current study are available from the corresponding author upon reasonable request.

## Abbreviations

CVI: Content validity index
CVR: Content validity ratio
HFA: Height-for-age
WFA: Weight-for-age
WFH: Weight-for-height
WHO: World Health Organization
